# Mortality of hospital or community-acquired Legionnaires’ disease (LD): a prospective study

**DOI:** 10.1186/1753-6561-5-S6-P306

**Published:** 2011-06-29

**Authors:** SP Cronenberger, C Chidiac, C Campese, D Che, S Jarraud, T Benet, P Vanheims, Study Group

**Affiliations:** 1 Infection Control Unit, Edouard Herriot Hospital, France; 2 Infectious and Tropical Diseases, Croix Rousse Hospital, Lyon, France; 3 National Institute for Public Health Surveillance, Saint Maurice, France; 4 French National Reference Center for Legionella, Bron, France

## Introduction / objectives

The clinical aspects and outcome of hospital (HA) and community-acquired (CA) cases of LD might be different. We explored these topics in 267 patients with LD hospitalized in France.

## Methods

20 HA cases and 247 CA, all being aged 60 and older infected with *Legionella pneumophila* serogroup 1 included in a French multicentric prospective study of LD (April/06-June/07) were compared. A confirmed HA case was defined when the entire incubation period (10 days, d) or more occurred during hospital stay, and a probable HA case was a patient hospitalized between 2d to 10d before onset. Cox regression was done to identify predictors of 30-days mortality (based on relative hazard [RH]) and survival probability was calculated using Kaplan-Meier method.

## Results

In the 20 HA, 12 (60%) were confirmed cases. No difference between the HA and CA groups was observed at admission for sex, age, smoking and alcohol habit, diabetes, respiratory, digestive, and neurological signs and intensive care unit (ICU) stay. Cancer/malignancy (45% [HA] *vs* 10% [CA], p<0.001), steroid therapy (35% *vs* 6%, p<0.001), *classified in* Fine score risk class IV-V (70% *vs* 47%, p=0.04), and mortality (45% *vs* 9%, p<0.001) were more prevalent in HA cases. The probability of survival at 30d was 50%, and 82% in HA and CA respectively (p<0.001). The RH of death for HA was 4.2 (95% CI 1.9-9.2; p<0.001) adjusted for age, ICU stay and renal failure.

## Conclusion

The risk of death was higher in HA LD. Severe underlying diseases might explain such outcome. These results emphasized the need of a prompt appropriate treatment when LD is suspected in hospitalized patients.

## Disclosure of interest

None declared.

